# Circulating T cell subsets are associated with clinical outcome of anti-VEGF-based 1st-line treatment of metastatic colorectal cancer patients: a prospective study with focus on primary tumor sidedness

**DOI:** 10.1186/s12885-019-5909-5

**Published:** 2019-07-15

**Authors:** Beatrix Bencsikova, Eva Budinska, Iveta Selingerova, Katerina Pilatova, Lenka Fedorova, Kristina Greplova, Rudolf Nenutil, Dalibor Valik, Radka Obermannova, Michael A. Sheard, Lenka Zdrazilova-Dubska

**Affiliations:** 1 Department of Comprehensive Cancer Care, Masaryk Memorial Cancer Institute, Brno, Czech Republic; 2 Regional Centre for Applied Molecular Oncology, Masaryk Memorial Cancer Institute, Brno, Czech Republic; 3 Department of Laboratory Medicine, Masaryk Memorial Cancer Institute, Brno, Czech Republic; 4 Department of Oncological and Experimental pathology, Masaryk Memorial Cancer Institute, Brno, Czech Republic

**Keywords:** Metastatic colorectal cancer, T cell subsets, Regulatory T cells, Antitumor immune response, Anti-VEGF, Primary colorectal carcinoma sidedness

## Abstract

**Background:**

In a prospective study with long-term follow-up, we analyzed circulating T cell subsets in patients with metastatic colorectal cancer (mCRC) in the context of primary tumor sidedness, *KRAS* status, and clinical outcome. Our primary goal was to investigate whether baseline levels of circulating T cell subsets serve as a potential biomarker of clinical outcome of mCRC patients treated with an anti-VEGF-based regimen.

**Methods:**

The study group consisted of 36 patients with colorectal adenocarcinoma who started first-line chemotherapy with bevacizumab for metastatic disease. We quantified T cell subsets including Tregs and CD8^+^ T cells in the peripheral blood prior to therapy initiation. Clinical outcome was evaluated as progression-free survival (PFS), overall survival (OS), and objective response rate (ORR).

**Results:**

1) mCRC patients with *KRAS* wt tumors had higher proportions of circulating CD8^+^ cytotoxic T cells among all T cells but also higher measures of T regulatory (Treg) cells such as absolute count and a higher proportion of Tregs in the CD4^+^ subset. 2) A low proportion of circulating Tregs among CD4^+^ cells, and a high CD8:Treg ratio at initiation of VEGF-targeting therapy, were associated with favorable clinical outcome. 3) In a subset of patients with primarily right-sided mCRC, superior PFS and OS were observed when the CD8:Treg ratio was high.

**Conclusions:**

The baseline level of circulating immune cells predicts clinical outcome of 1st-line treatment with the anti-VEGF angio/immunomodulatory agent bevacizumab. Circulating immune biomarkers, namely the CD8:Treg ratio, identified patients in the right-sided mCRC subgroup with favorable outcome following treatment with 1st-line anti-VEGF treatment.

**Electronic supplementary material:**

The online version of this article (10.1186/s12885-019-5909-5) contains supplementary material, which is available to authorized users.

## Background

Immune cells play a crucial role in control of tumor growth, potentially leading to elimination of cancer cells even while immunosuppression contributes to evasion by malignant cells. Cytotoxic CD8^+^ T cells (CTLs) represent one of the most important effectors of anti-cancer immunity [1]. Accumulation of CD8^+^ cells in solid tumors of various origins including colorectal carcinoma [2–6] has been associated with favorable prognosis and has led to definition of the immunoscore concept that is now emerging in clinical practice in the management of colorectal cancer [7, 8].

Regulatory T cells (Tregs) prevent immune hypersensitivity and extensive inflammatory responses. However, through their immunosuppressive properties, Tregs can contribute to escape of tumor cells from immune surveillance [9]. A connection between a high number of Tregs and worse prognosis has been described in several tumor types (reviewed in [10]). There are at least two major subsets of Tregs; natural Treg cells (nTregs) that are generated in the thymus and are constitutively present in blood and lymphoid organs, and induced (or inducible) Tregs (iTregs) that develop outside of the thymus from naïve T cells during immune responses [9]. nTregs can be recognized by their CD4^+^ CD25^+^ FoxP3^+^ CD127^low/−^ neuropilin^+^ surface immunophenotype [9, 11]. In cancer patients, Tregs can be detected in both the peripheral blood circulation and in the tumor microenvironment (TME), although mechanisms regulating the homing of Tregs into and from the TME are not yet fully elucidated. Nevertheless, in colon cancer patients, cancer-associated circulating Tregs have been shown to inhibit proliferation of autologous T cells [12] and effector T cell migration into tumors through an adenosine-dependent mechanism [13]. Moreover, the TME and gut microbiome contribute to Treg plasticity and heterogeneity [14, 15] and also consequently to the differential prognostic role of Tregs in colorectal cancer [16–18]; for example, in the context of primary colorectal cancer, Tregs may play both an anti-inflammatory and also a potentially anti-cancer role. In metastatic CRC, as well as other cancer types including breast cancer [19], pancreatic cancer [20], and head-and-neck squamous cell cancer [21], elevated numbers of circulating Tregs may be related to worse prognosis.

CRC is a heterogeneous disease that develops through different molecular pathways affecting distinct gene expression, tumor and TME phenotype, and tumor behavior [22–25]. Consensus molecular subtype (CMS) numbers 1–4 have been associated with distinct immune characterization, as 1) immune activated, highly immunogenic CMS1 tumors of hypermutated microsatellite instable origin with increased infiltration of immune effector cells into the TME [26–28], 2) canonical CMS2 and metabolic CMS3 subtypes which are generally immune-ignorant, and 3) mesenchymal CMS4 tumors with inflamed, immune-tolerant TMEs representing the subtype with dominant immunosuppressive features (TGF-β, myeloid-derived suppressor cells / MDSC, Tregs, Th17).

Metastatic colorectal cancer is an incurable disease treated in a palliative setting by chemotherapy or chemotherapy plus the anti-VEGF antibody bevacizumab as a tumor angiogenesis modifying agent. Median progression-free survival is reported to be 11.5 months and median overall survival is 29.5 months from initiation of first line (1st-line) therapy with bevacizumab and chemotherapy [29]. Together with its angiomodulatory properties, bevacizumab may influence immune parameters including cells of the adaptive immune response. Bevacizumab partially reversed VEGF-induced inhibition of dendritic cell development [30, 31] and VEGF-associated increases in Tregs [32]. It has also been reported that bevacizumab can directly decrease the level of Tregs and impair their function via VEGF receptors expressed on the surface of Tregs [33]. Finally, bevacizumab-based therapy was shown to increase circulating B and T cells and these effects were associated with better clinical outcome in mCRC [34].

In a prospective study, we analyzed circulating T cell subsets in patients with metastatic colorectal cancer in the context of primary tumor sidedness, *KRAS* status, and clinical outcome. Our primary goal was to investigate whether baseline levels of circulating immune cells could be a potential biomarker of the clinical outcome of mCRC patients treated with an anti-VEGF-based regimen.

## Methods

### Study group

**Fig. 1:**
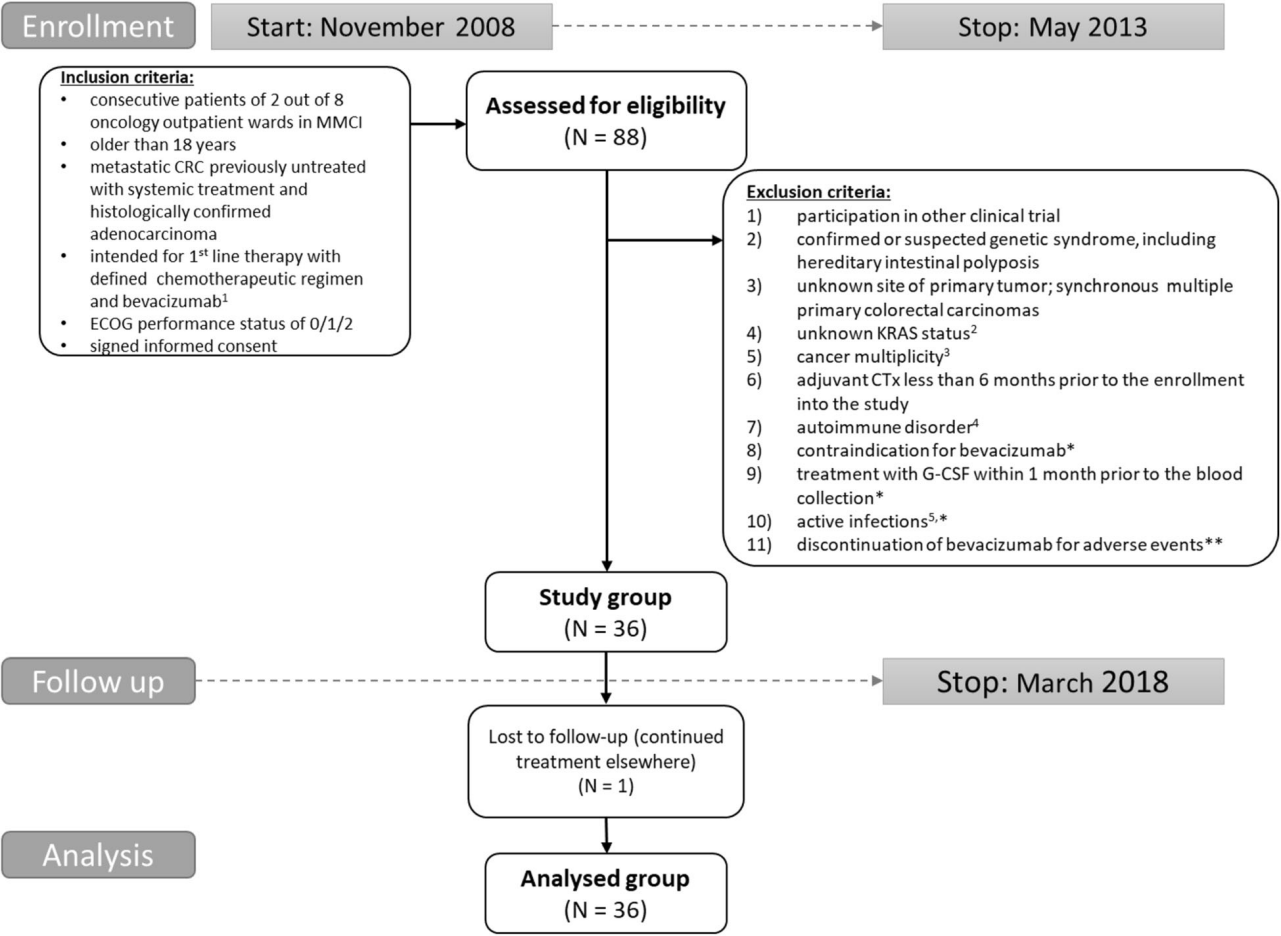
Study group definition. ^**1**^ Intended CTx regimen was chosen from among the following: CapeOX (oxaliplatin 130 mg/m^2^ IV day 1, capecitabine 1000 mg/m^2^ twice daily per os (PO) for 14 days, repeat every 3 weeks); CapeIRI (irinotecan 250 mg/m^2^ day 1, capecitabine 1000 mg/m^2^ twice daily PO for 14 days, repeat every 3 weeks); FOLFOX4 (oxaliplatin 85 mg/m^2^ intravenous (IV) day 1, Leucovorin 200 mg/m^2^ IV days 1 and 2, 5- fluorouracil 400 mg/m^2^ IV bolus on day 1 and 2, 5- fluorouracil 600 mg/m^2^ 22-h continuous infusion days 1 and 2, repeat every 2 weeks); FOLFIRI (irinotecan 180 mg/m^2^ IV day 1, Leucovorin 400 mg/m^2^ IV day 1, 5- fluorouracil 400 mg/m^2^ IV bolus day 1, then 5- fluorouracil 1200 mg/m^2^ /d continuous infusion days 1 and 2, repeat every 2 weeks). Bevacizumab was administered on the first day of each cycle at a dose of 5 mg/kg IV in combination with the 2-week regimen and at a dose of 7.5 mg/kg IV with the 3-week regimen. ^**2**^ *KRAS* status was not tested (not yet performed or not ordered during the enrollment period) for mCRC patient management; *KRAS* testing was performed by ISO 15189-accredited methods; specifically 2008 - December 2011 by real time PCR method using TheraScreen (DxS); January 2012 – May 2013 using the Cobas® KRAS Mutation Test (Roche Diagnostics). ^**3**^ prior malignancy except for locally curable cancers such as basal or squamous cell skin cancer, superficial bladder cancer, or carcinoma in situ of the prostate, cervix, or breast, curatively treated with no evidence of disease for ≥3 years. ^**4**^ active, known, or suspected autoimmune disease requiring systemic treatment with immunosuppressive medication including chronic inflammatory bowel disease (Crohn’s disease or ulcerative colitis). ^**5**^ active infection at the time of blood collection including clinically significant non-healing or healing wound, ulcer. * exclusion criterion applicable if appears before the blood collection. ** exclusion criterion applicable if appears before the achievement of objective clinical response

The prospective study group consisted of 36 patients with histologically confirmed *KRAS*-tested metastatic adenocarcinoma of colon or rectum who began 1st-line treatment for metastatic disease between November 2008 and May 2013. A flow chart of patient enrollment with detailed inclusion and exclusion criteria is shown in Fig. 1. Briefly, consecutive patients were older than 18 years, had an Eastern Cooperative Oncology Group performance status of 0/1/2, and signed inform consent. Exclusion criteria were: known alteration of immune system (active infections or autoimmune disorder); treatment with G-CSF; contraindication to treatment with bevacizumab or its discontinuation; prior chemotherapy (CTx) for advanced disease, or adjuvant CTx less than 6 months before enrollment onto study, cancer multiplicity. Choice of chemotherapy regimen was at the physicians’ discretion. Bevacizumab was administered at a dose of 5 mg/kg IV with the 2-week regimen or at a dose of 7.5 mg/kg IV with the 3-week regimen. Patients’ responses to treatment and tumor measurements were evaluated with computer tomography scan by a staff radiologist according to RECIST criteria. PFS was defined as the time from the beginning of treatment until the first observation of disease progression or death from any cause, while OS was defined as the time from the beginning of treatment until death from any cause. Patients were followed-up until death or loss to follow-up. Survival rates were last updated in March 2018. ORR was defined as the proportion of patients who have a partial or complete response to treatment. Baseline characteristics of patients are summarized in Additional file 1: Table S1.

### Sample collection and lymphocyte count evaluation

Peripheral blood specimens were collected at initiation of anti-VEGF treatment in a 2.6 mL S-Monovette® tube with K_3_EDTA anticoagulant (Sarstedt, catalog number 04.1901) in a phlebotomy room in close proximity to the laboratory where analysis was performed. Blood specimens were mixed for several minutes on a roller mixer. Immediately after that, absolute lymphocyte count was obtained from the complete blood count by a differential analyzer Sysmex XE 5000 (Sysmex Corporation, Japan). Absolute lymphocyte count was used for calculation of the absolute count of T cell subsets.

### Flow cytometry – T cell subset quantification

Lymphocyte subsets were evaluated within 3 h of blood collection. For Treg detection as CD3^+^CD4^+^CD25^+^CD127^−/low+^ cells and CD4^+^ T cell detection, 50 μL of whole blood was stained with a premixed cocktail of conjugated mAbs (Beckman Coulter) for the following markers, CD3-FITC (clone UCHT1), CD25-PC5 (clone B1.49.9), CD4-PC7 (clone 13B8.2), and CD127-PE (clone R34.34) in concentrations according to manufacturer instructions. The gating strategy for CD3^+^CD4^+^CD25^+^CD127^−/low+^ cells including details on gating set-up and the analytical and statistical comparability of CD25^+^CD127^−/low+^ and CD25^+^FoxP3^+^ quantification approaches are shown in Additional file 1: Figure S1. CD8^+^ cells were detected using 50 μL of whole blood stained with tetraCHROME CD45-FITC/CD4-PE/CD8-ECD/CD3-PC5 multi-color reagent (Beckman Coulter) in concentrations according to the manufacturer instructions. After a 15 min staining for Tregs or CD8^+^ T-cells in the dark, red blood cells were lysed for 15 min in the dark by adding 600 μL of VersaLyse Lysing Solution (Beckman Coulter, France). Cells were subsequently analyzed using a Cytomics FC 500 flow cytometer, hardware compensation and CXP software (Beckman Coulter, USA).

### Statistical analysis

Wilcoxon two-sample two-tailed test was used to compare continuous variables between the two groups in the Results section, part I. Survival probabilities were estimated using the Kaplan-Meier method in the Results section part II and III. Log-rank test was used to assess the association of categorical variables with survival endpoints. Hazard ratios were determined using Cox proportional hazard model. Logistic regression was used to predict objective responses and to determine odds ratio. The need for adjustment by common biomarkers was considered in the Results section part II and III. The Cox model with interaction term was used to compare effects in subgroups in the Results section part III. Optimal cut points of continuous variables with respect to the survival endpoints were determined using the conditional hazard function which was estimated using smoothing techniques based on kernel methods [35]. Statistical comparison of two Treg quantification approaches was performed using Bland-Altman plot and Passing-Bablok regression in MS Excel. Conditional hazard functions were estimated in MATLAB, other analyses were performed in R, a language and environment for statistical computing (R Core Team, 2013). Results with *p* < 0.05 were considered statistically significant.

## Results

### Circulating Tregs, CD8^+^ CTLs and CD8:Treg ratio in metastatic colorectal cancer patients in the context of primary tumor sidedness and *KRAS* status

**Fig. 2:**
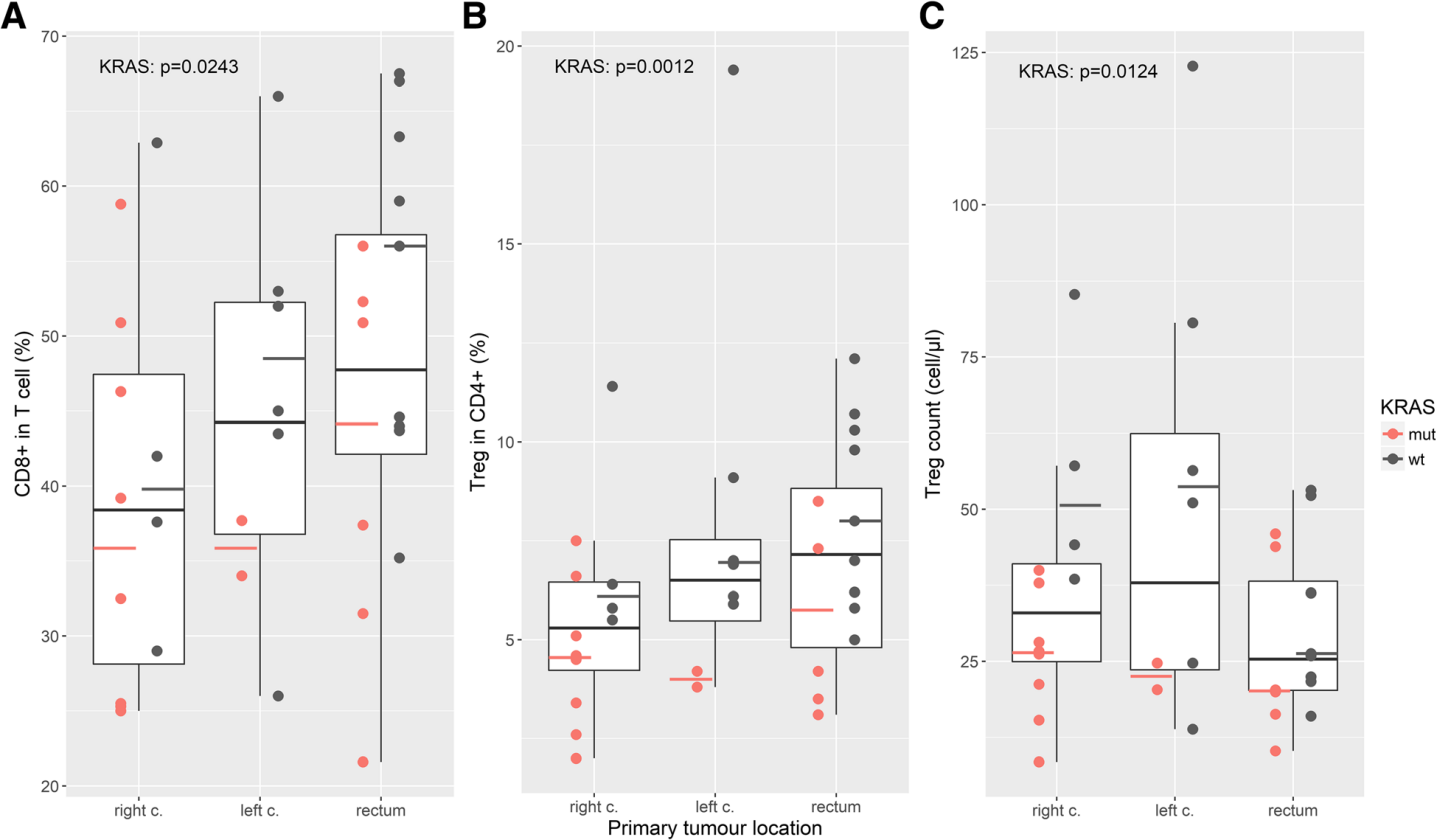
Circulating CTLs and Tregs in metastatic colorectal cancer patients in the context of primary tumor sidedness and *KRAS* mutation. *p*-values refer to the level of circulating T cell subsets in *KRAS* wt vs. *KRAS* mut in the entire study group

Relative and absolute numbers of circulating immune cells were quantified in mCRC patients at the initiation of 1st line anti-VEGF-based therapy and were evaluated in the context of primary tumor sidedness and *KRAS* status. Regardless of primary tumor sidedness, there was no difference in circulating Treg or CD8^+^ CTL count. A trend was observed toward an increasing proportion of CD8^+^ CTLs in T cells from proximal to distal tumor locations. Notably, *KRAS* wt colorectal cancers exhibited a significantly higher proportion of CD8^+^ CTLs among T cells but also higher Treg measures (absolute count and the proportion of Tregs among CD4^+^ cells (Table 1, Fig. 2).

**Table 1:** Medians of circulating immune cells in mCRC patient subgroups

	mCRC	Primary tumor location			*KRAS* status		
		right c.	left c.	r.s./rectum	*KRAS* wt	*KRAS* mut	
Lymphocytes (cells/μL)	1445	1593	1469	1309	1521		1312
CD3^+^ in lymphocytes (%)	63	65	71	59	64		65
T cell count (cells/μL)	1042	1137	1151	894	1220		894
CD8^+^ in T cells (%)	44	38	44	48	45	*	38
CD8^+^ count (cells/μL)	380	372	511	401	558		309
Treg in lymphocytes (%)	1.9	1.7	2.0	2.0	2.3		1.7
Treg in CD4^+^ (%)	6.2	5.3	6.5	7.2	7.0	**	4.4
Treg count (cells/μL)	26.5	33.0	37.9	25.4	38.5	*	23.0
CD8:Treg	13.1	10.9	13.3	15.7	11.5		14.0

Stars indicate statistically significant difference in mCRC patients between respective subgroups: *p < 0.05, ** *p* < 0.005. c, colon; r.s., rectosigma

### Circulating Tregs, CD8^+^ CTLs, CD8:Treg ratio, and clinical outcome of 1st-line anti-VEGF-based therapy of mCRC

**Fig. 3:**
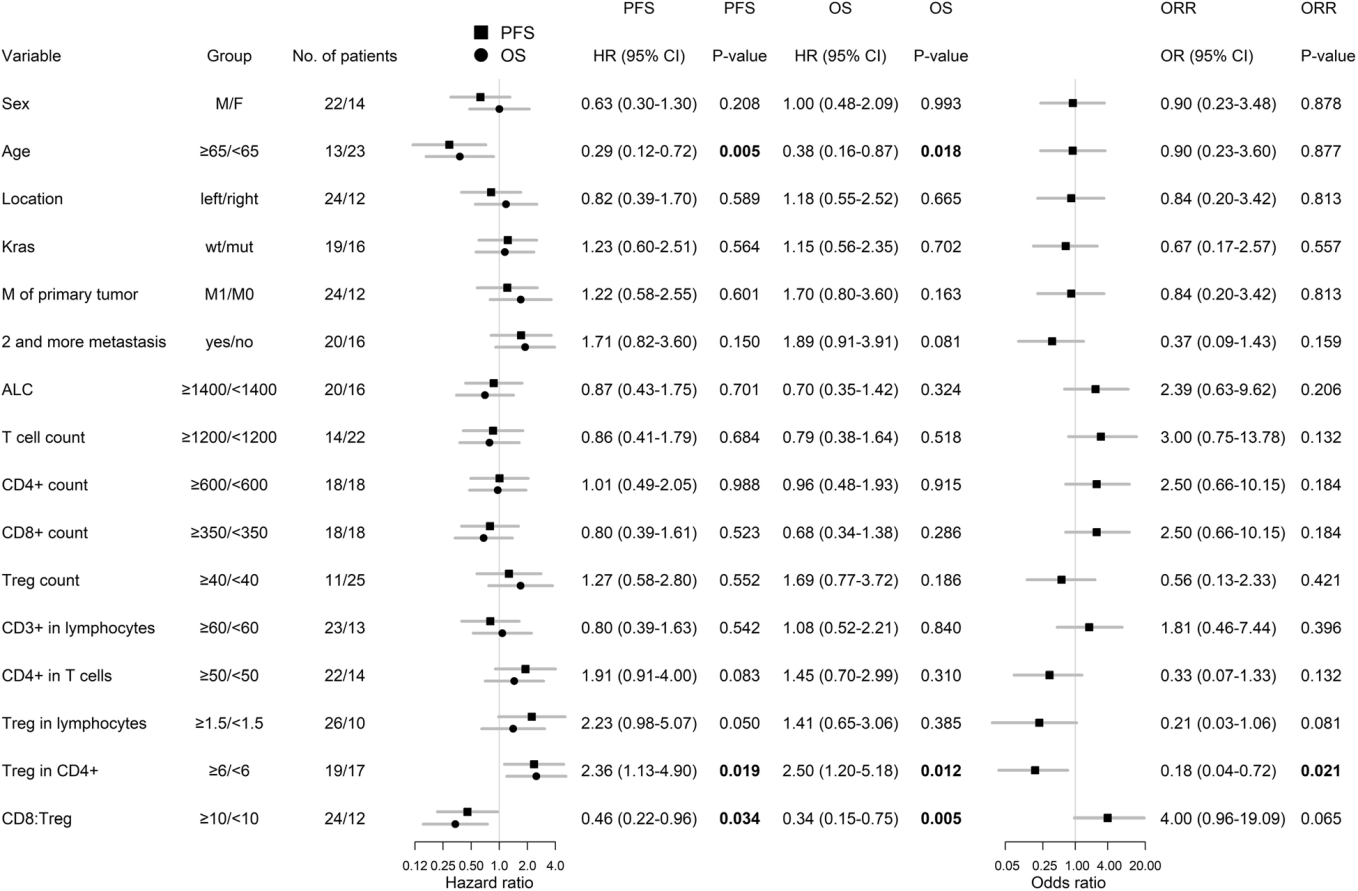
Results of univariable analysis for progression-free, overall survival and objective response rate (ORR). Location: “right” = right colon, “left” = left colon and rectum. ALC = absolute lymphocyte count

Median length of follow-up was 77.4 months. Median PFS for the study group was 10.5 months (95% CI: 8.8–16.3 months), median overall survival was 30.0 months (95% CI: 23.3–38.5 months), and ORR was 55.6% (95% CI: 39.6–70.5%). Survival and response rate analysis was performed for parameters clinically relevant for metastatic colorectal cancer, such as gender, age, M0 vs. M1, number of metastatic sites, *KRAS* status, and primary tumor sidedness (Fig. 3). Of those, age < 65 years was associated with shorter PFS and OS but not ORR (Fig. 3). Levels of circulating immune cells at 1st-line anti-VEGF therapy initiation were investigated in the context of clinical outcome using the conditional hazard function estimated by smoothing techniques (Additional file 1: Figure S2). Cut-off levels for each parameter, dividing cases to “low” and “high”, were established as shown in Additional file 1: Figure S2 and subgroups defined by levels of immune parameters were analyzed for PFS and OS (Fig. 3). Of those, the baseline proportion of Tregs in CD4^+^ cells was predictive for shorter PFS and OS and worse ORR, and the baseline CD8:Treg ratio was predictive for longer PFS and OS. In the subgroup of mCRC patients with < 6% frequency of Tregs among CD4^+^ cells, median PFS (mPFS) was 16.2 months, mOS was 38.5 months, and ORR was 76.4% compared to those with a high frequency of circulating Tregs of ≥6% among CD4^+^ cells which had a mPFS of 8.8 months, mOS of 22.3 months, and ORR of 36.8%. In the subgroup of mCRC patients with a high CD8:Treg ratio of ≥10, mPFS was 12.6 months and mOS was 37.8 months compared to those with a ratio of circulating CD8:Treg of < 10 which had an mPFS of 8.1 months and mOS of 21.0 months (Additional file 1: Table S2).

### Circulating Tregs, CD8^+^ CTLs and CD8:Treg ratio and the clinical outcome of anti-VEGF-based therapy of mCRC in the context of primary tumor sidedness

**Fig. 4:**
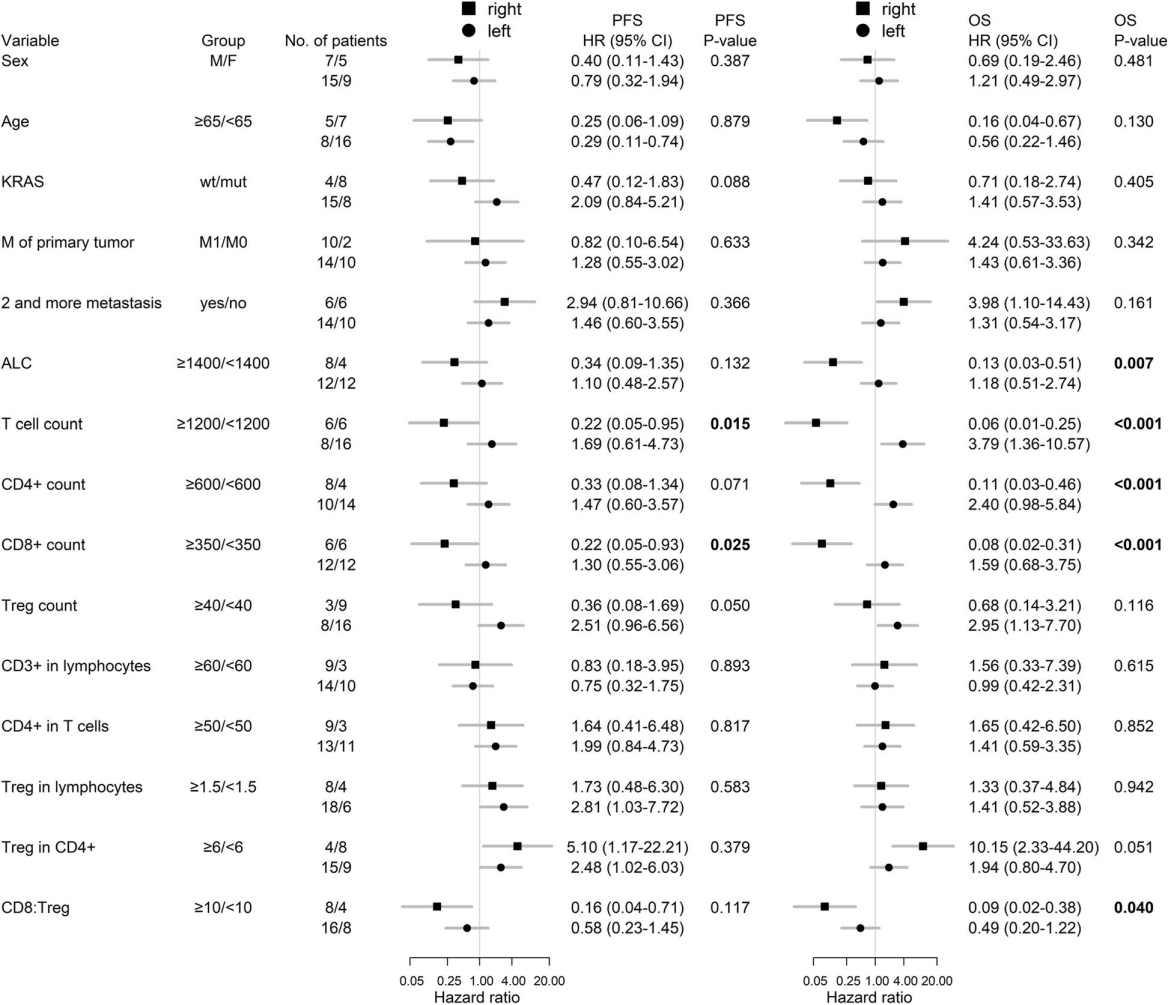
Results of Cox analyses for progression-free and overall survival according to primary tumor location. P-values correspond to test significance of the interaction term (test of different effects of variables according to primary right- and left-sided mCRC). Location: “right” = right colon, “left” = left colon and rectum

The association between number of circulating immune cells and clinical outcome of mCRC therapy was further analyzed in the context of primary tumor sidedness (Fig. 4). The predictive value of the baseline proportion of Tregs among CD4^+^ cells and the CD8:Treg ratio had the same direction in primary right- and left-sided mCRC. In addition to the strong association between high CD8:Treg ratio and favorable clinical outcome in the entire study group, the association between high CD8:Treg ratio and longer overall survival was significantly higher in primary right-sided mCRC (Fig. 4, Additional file 1: Figure S3) and those with a high CD8:Treg ratio of ≥10 had a mPFS of 14.4 months and a mOS of 39.9 months compared to those with a low ratio of circulating CD8:Treg of < 10 which had a mPFS 7.1 months and a mOS of 12.9 months (Additional file 1: Table S2). In the subgroup of mCRC patients with primary tumors in the right colon, a significant interaction between primary tumor sidedness and the predictive value of absolute T cell count as well as the absolute CD8^+^ and CD4^+^ cell counts revealed an association of poor PFS and OS with low baseline circulating absolute T cells or CD8^+^ CTLs (Fig. 4, Additional file 1: Table S2 and Figure S3).

## Discussion

Here we show that the baseline level of parameters derived from circulating Tregs, namely the Treg proportion among CD4^+^ T cells and the CD8:Treg ratio, at the initiation of anti-VEGF-based therapy predicts treatment outcome in terms of both PFS and OS, and objective response rate. Our findings are in agreement with a study by Roselli et al. by showing that a low baseline proportion of Tregs in PBMC, but not any other clinical or laboratory parameter evaluated, is associated with favorable outcome in mCRC patients receiving 1st-line FOLFIRI plus bevacizumab [36]. Roselli et al. emphasized the unexplained lack of association between clinical outcome and CD8^+^ T cells [36] that we also observed when baseline circulating immune parameters from mCRC patients were analyzed irrespective of primary tumor sidedness. Nevertheless, and based on our previous findings of poor clinical outcome of mCRC patients with primary tumors in the right colon [37] and the differential impact of *KRAS* status for 1st-line anti-VEGF-based therapy in primary right vs. left-sided mCRC [38], we analyzed circulating immune cells in the context of primary tumor sidedness, revealing that the association of previously identified Treg-associated biomarkers, as well as a baseline number of circulating CD8^+^ T cells, with clinical outcome of 1st-line anti-VEGF-based therapy is particularly strong in mCRC patients with primary tumor in the right colon.

The differential disease behavior of primarily right vs. left-sided mCRC is substantiated by the prevalence of distinct colorectal cancer subtypes within the colon and rectum [39]. Based on the association of the immune-activated, highly immunogenic CMS1 tumor subtype with right-sided tumor location [39] on the one hand, and the strong association of favorable circulating immune signature (low Tregs, high CD8^+^ T cells, high CD8:Treg ratio) and favorable clinical outcome in primary right-sided mCRC on the other, we propose that right-sided mCRC patients with favorable circulating immune signature overlap with a subgroup of patients with immune-activated tumors that clearly benefit from immunomodulatory anti-VEGF-based therapy. Our hypothesis that immune characteristics in the TME are reflected in the circulation is further supported by the finding of an association of *KRAS* mutant status with reduction in both CD8^+^ T cell count and number of Tregs. CMS2 and 3 subtypes are associated with reduced immune infiltration and reactivity, and this immune quiescence is more profound in *KRAS*-mutated tumors [40] and is likely mirrored in peripheral blood.

Due to the small size of study group, the cut-off levels of immune cells stratifying prognostic subgroups may not be accurate and should be validated in larger cohort of patients. Limited size of the study group also did not allow multivariable analysis. A strength of this study is its long-term follow-up. On the other hand, during the time period when the study was designed, biomarkers such as *NRAS*, *BRAF*, and MSI were just emerging in the clinical practice of colorectal cancer patient management and unfortunately were not analyzed in the context of circulating immune cells in mCRC treatment with bevacizumab. Thus, it remains to be investigated whether the subset of patients with right-sided tumor and favorable circulating immune signature overlaps with the MSI-H/CMS1 subset and may therefore be a good candidate for immunotherapy with checkpoint inhibitors. Also, it remains to be addressed whether mCRC patients, particularly those with right-sided tumors with an immunosuppressive circulating immune signature (high Tregs, low CD8^+^ T cells and/or low CD8:Treg ratio) would benefit from the aggressive, triple combination chemotherapy regimen FOLFOXIRI [41].

## Conclusions

Circulating immune parameters derived from the baseline level of CD8^+^ CTLs and Tregs may predict clinical outcome following 1st-line treatment with the anti-VEGF angio/immunomodulatory agent bevacizumab and thereby identify mCRC patients, particularly within the primarily right-sided subgroup, who have favorable outcome.

## Additional files

Additional file 1: **Table S1.** Baseline characteristics of mCRC patients included in the study. **Figure S1.** Gating strategy for CD3^+^CD4^+^CD25^+^CD127^−/low+^ cells and the analytical comparability of a) CD25^+^CD127^−/low+^ and b) CD25^+^FoxP3^+^ quantification approaches. Statistical comparison of these approaches using c) Bland-Altman plot and d) Passing-Bablok regression. **Figure S2.** Determination of the optimal cut points for circulating immune cells with respect to PFS and OS using kernel estimates of conditional hazard functions. **Table S2.** Characteristics of clinical outcome (PFS and OS), proportion of Tregs in the CD4^+^ cell subset, and the CD8: Treg ratio. **Figure S3.** Circulating immune cells and clinical outcome of anti-VEGF-based therapy of mCRC in the context of primary tumor sidedness. (DOCX 2640 kb)

Additional file 2: Spreadsheet with data generated and analyzed during the study. (XLSX 20 kb)

## Data Availability

All data generated and analysed during this study are included in this published article (Additional file 2).

## Abbreviations

ALC: absolute lymphocyte count
CMS: Consensus molecular subtype
CR: complete remission
CTLs: Cytotoxic CD8^+^ T cells
CTx: chemotherapy;
iTregs: induced (or inducible) Tregs
IV: intravenous
mCRC: metastatic colorectal cancer
NA: Not Available
NS: not specified
nTregs: natural Treg cells
ORR: objective response rate
OS: overall survival
PD: progressive disease
PFS: progression-free survival
PO: per os
PR: partial remission
PS: performance status
SD: stable disease
TME: tumor microenvironment
Tregs: Regulatory T cells
