# Supplementary material for: Circulating T cell subsets are associated with clinical outcome of anti-VEGF-based 1st-line treatment of metastatic colorectal cancer patients: a prospective study with focus on primary tumor sidedness
Source: BMC Cancer. 2019 Jul 15;19:687. doi: 10.1186/s12885-019-5909-5 (PMC6631500; doi:10.1186/s12885-019-5909-5)
Supplement: Supplementary file 1 — Table S1. Baseline characteristics of mCRC patients included in the study. Figure S1. Gating strategy for CD3+CD4+CD25+CD127−/low+ cells and the analytical comparability of a) CD25+CD127−/low+ and b) CD25+FoxP3+ quantification approaches. Statistical comparison of these approaches using c) Bland-Altman plot and d) Passing-Bablok regression. Figure S2. Determination of the optimal cut points for circulating immune cells with respect to PFS and OS using kernel estimates of conditional hazard functions. Table S2. Characteristics of clinical outcome (PFS and OS), proportion of Tregs in the CD4+ cell subset, and the CD8: Treg ratio. Figure S3. Circulating immune cells and clinical outcome of anti-VEGF-based therapy of mCRC in the context of primary tumor sidedness. (DOCX 2640 kb) [file 12885_2019_5909_MOESM1_ESM.docx]

**Table S1:** Baseline characteristics of mCRC patients included in the study.

| \| Patients included in the study \| n = 36 \| \| --- \| --- \| |
| --- | --- | --- |
| \| Male; n (%) \| 22 (61 %) \| \| --- \| --- \| |
| \| Age at time of diagnosis; median, (min-max) \| 62 (32-78) \| \| --- \| --- \| |
| \| ECOG performance status at 1^st^-line treatment; n (%)  PS 0  PS 1  PS 2 \| 10 (28 %)  24 (67 %)  2 (5 %) \| \| --- \| --- \| |
| \| Site of primary tumor  Colon; n (% of all)  Cecum; n (% of primary tumor in colon)  Ascending colon; n (% of primary tumor in colon)  Hepatic flexure of colon; n (% of primary tumor in colon)  Transverse colon; n (% of primary tumor in colon)  Splenic flexure of colon; n (% of primary tumor in colon)  Descending colon; n (% of primary tumor in colon)  Sigmoid colon; n (% of primary tumor in colon)  Rectosigma; n (% of all)  Rectum; n (% of all) \| 20 (55 %)  2 (10 %)  6 (30 %)  3 (15 %)  1 (5 %)  1 (5 %)  1 (5 %)  6 (30 %)  6 (17 %)  10 (28 %) \| \| --- \| --- \| |
| \| Histology  Adenocarcinoma; n (% of all)  Grade 1; n (% of adenocarcinomas)  Grade 2; n (% of adenocarcinomas)  Grade 3; n (% of adenocarcinomas)  Grade NS; n (% of adenocarcinomas)  Mucinous adenocarcinoma; n (% of all) \| 32 (89 %)  2 (6 %)  18 (57 %)  9 (28 %)  3 (9 %)  4 (11 %) \| \| --- \| --- \| |
| \| *KRAS*, n (% of all) = data available  Wild-type; n (% of examined)  Mutant; n (% of examined) \| 35 (97 %)  19 (54 %)  16 (46 %) \| \| --- \| --- \| |
| \| Location of metastatic sites; n (%)  Liver  Lung  Peritoneum/Mesenterium  Lymph nodes  Bones  Other \| 22 (61 %)  9 (25 %)  9 (25 %)  18 (50 %)  1 (3 %)  1 (3 %) \| \| --- \| --- \| \| Primary tumor resection; n (% of all) \| 32 (89 %) \| \| Adjuvant / neoadjuvant chemotherapy; n (% of all) \| 12 (33 %) \| \| Adjuvant radiotherapy or chemoradiotherapy; n (% of all) \| 10 (28 %) \| |
| \| 1st line treatment; n (%)  CapeOX+ bevacizumab  FOLFOX + bevacizumab  CapeIRI+ bevacizumab \| 32 (89 %)  3 (8 %)  1 (3 %) \| \| --- \| --- \| \| Best objective response \|  \| \| SD  PR  CR  PD \| 16 (44 %)  14 (39 %)  6 (17 %)  0 (0 %) \| |

Note: PS = performance status; NS = not specified; SD = stable disease; PR = partial remission; CR = complete remission; PD = progressive disease.

**Fig. S1:** Gating strategy for CD3^+^CD4^+^CD25^+^CD127^-/low+^ cells and the analytical comparability of a) CD25^+^CD127^-/low+^ and b) CD25^+^FoxP3^+^ quantification approaches. Statistical comparison of these approaches using c) Bland-Altman plot and d) Passing-Bablok regression.


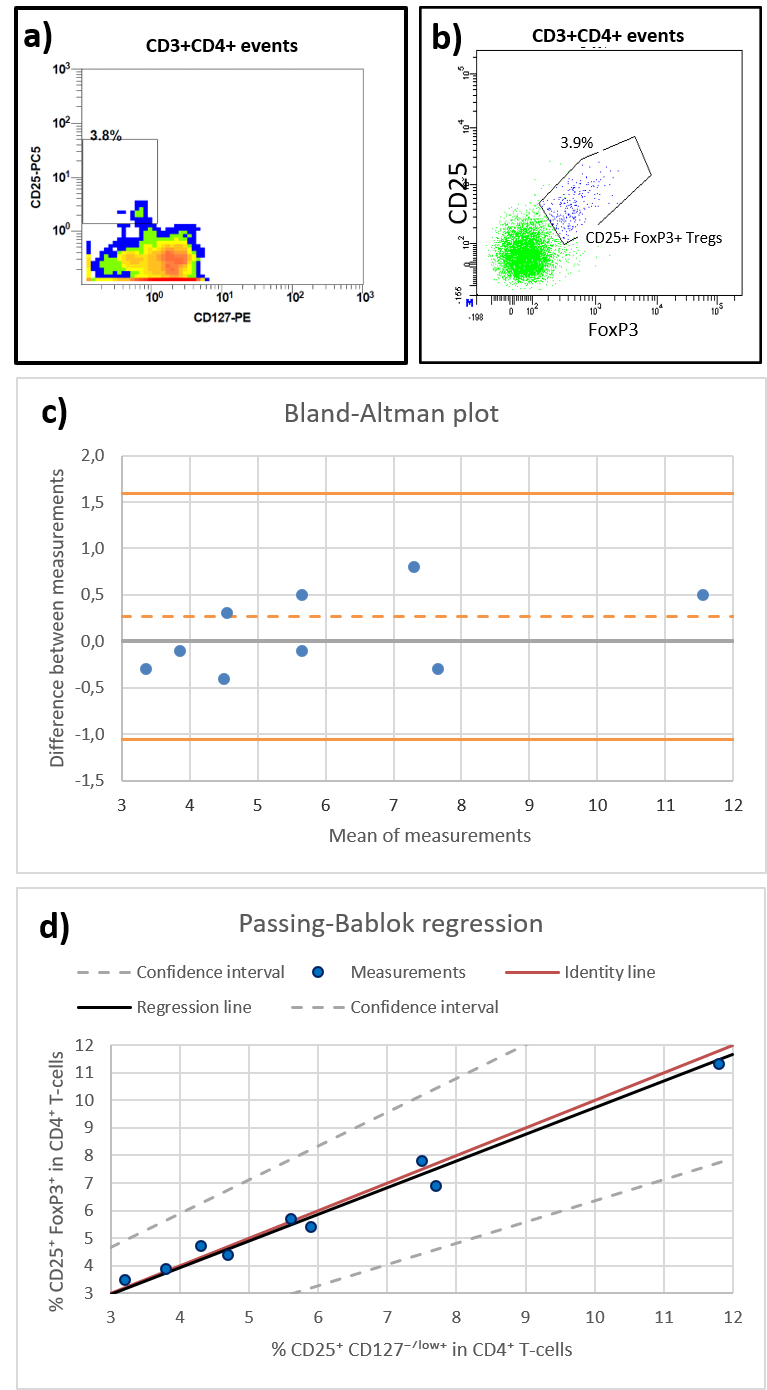


Legend: Using 10 control samples of peripheral blood, Tregs were quantified by two approaches as CD4^+^ CD25^+^ FoxP3^+^ cells using surface staining for CD4 and CD25 and intracellular staining for FoxP3, and as CD4^+^ CD25^+^ CD127^-/low+^ cells using surface staining. Gating strategy for **a)** CD25^+^ CD127^-/low+^ in CD4^+^ and for **b)** CD25^+^ FoxP3^+^ in CD4^+^ staining is shown for a representative measurement of a control sample. Gating for CD25 positivity was set-up using IgG2-PC5 isotype control (clone 7T4-1F5). Proportions of Tregs quantified as CD25^+^ CD127^-/low+^ in CD4^+^ cells did not statistically differ from Treg quantification using standard approach detecting Tregs as CD25^+^ FoxP3^+^ cells in CD4^+^ lymphocytes as shown on **c)** Bland-Altman plot and **d)** Passing-Bablok regression.

**Fig. S2:** Determination of the optimal cut points for circulating immune cells with respect to PFS and OS using kernel estimates of conditional hazard functions.

**
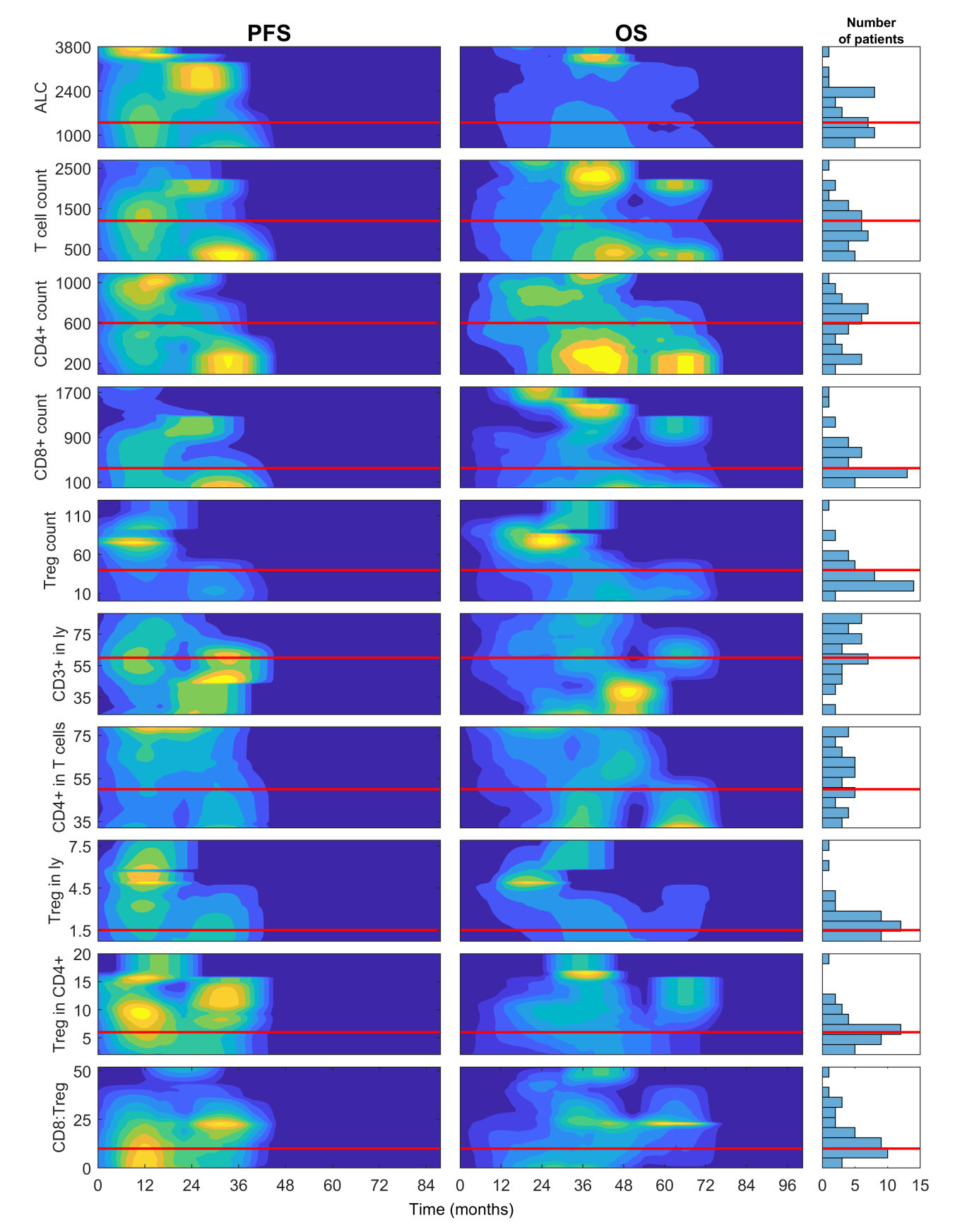
**

Legend: Contour plots of kernel estimates of conditional hazard functions with circulating immune cells as covariates are shown for PFS in the first column and OS in the second column with yellow areas representing the highest hazard. Histograms in the third column detail the number of patients with a respective circulating immune cell levels. The red lines represent identified cut-offs.

**Table S2:** Characteristics of clinical outcome (PFS and OS), proportion of Tregs in the CD4^+^ cell subset, and the CD8:Treg ratio.

|  |  |  |  |  | **mPFS** (95% CI) months |  | **mOS** (95% CI)  months |
| --- | --- | --- | --- | --- | --- | --- | --- |
| Treg in CD4^+^ | <6 | All | | 16.2 (11.0,35.6) | | 38.5 (29.2 63.0) | |
|  |  |  | Right |  | 14.4 (10.5, NA) |  | 39.9 (29.2, NA) |
|  |  |  | Left |  | 20.2 (11.0, NA) |  | 37.8 (28.4, NA) |
|  | ≥6 | All | | 8.8 (8.1,15.7) | | 22.3 (18.3,35.2) | |
|  |  |  | Right |  | 8.5 (4.8, NA) |  | 15.7 (12.2, NA) |
|  |  |  | Left |  | 9.7 (8.0, 29.0) |  | 27.9 (20.7, 43.5) |
| CD8:Treg | <10 | All | | 8.1 (6.6, NA) | | 21.0 (13.6, NA) | |
|  |  |  | Right |  | 7.1 (7.8, NA) |  | 12.9 (12.2, NA) |
|  |  |  | Left |  | 11.4 (8.0, NA) |  | 26.7 (20.7, NA) |
|  | ≥10 | All | | 12.6 (9.7, 27.9) | | 37.7 (28.4, 51.3) | |
|  |  |  | Right |  | 14.4 (10.5, NA) |  | 39.9 (29.2, NA) |
|  |  |  | Left |  | 11.0 (9.7, NA) |  | 33.0 (26.1, 51.3) |

NA = Not Available.

**Fig. S3:** Circulating immune cells and clinical outcome of anti-VEGF-based therapy of mCRC in the context of primary tumor sidedness.

Note: CD8+ counts are per μL.
